# Impulse control disorders and use of dopamine agonists in early onset Parkinson’s disease

**DOI:** 10.3389/fneur.2024.1404904

**Published:** 2024-05-22

**Authors:** Pierpaolo Turcano, Jessie Jacobson, Khaled Ghoniem, Aidan Mullan, Emanuele Camerucci, Cole Stang, Capucine Piat, James H. Bower, Rodolfo Savica

**Affiliations:** ^1^Department of Neurology, Mayo Clinic, Rochester, MN, United States; ^2^Department of Neurology, University of Kansas Medical Center, Kansas City, KS, United States

**Keywords:** early-onset Parkinson’s disease, impulse control disorder, dopamine agonists, Parkinson, Parkinson therapy

## Abstract

**Introduction:**

Impulse control disorders (ICDs) are defined as excessive and repetitive behaviors that may affect Parkinson’s disease (PD) patients exposed to dopamine agonists. Current data on ICDs in patients with early-onset Parkinson’s disease (EOPD) is lacking. In this study we aim to assess the frequency of use of dopamine agonists, the prevalence of ICDs, and to explore potential factors associated with their development in patients with EOPD.

**Methods:**

We used the Mayo Clinic Data Explorer system to investigate a population-based cohort of EOPD patients between 1990 and 2022 at Mayo Clinic, Rochester, MN. We used ICD coding for parkinsonism; then, we reviewed all the clinical records and included only those patients with a clinical diagnosis of PD with symptoms onset at or before the age of 50, and who developed ICDs after using therapeutic doses of dopamine agonists.

**Results:**

A total of 831 (513 males and 318 females) patients with EOPD were included with a median age at symptom onset of 42 years of age (CI: 37–46). Dopamine agonists were used in 49.7% of all patients; of these, only 14.5% developed symptoms of one or more ICDs. Hypersexuality was the most commonly observed ICD (38.3%), and the only one having a statistically significant male predominance (*p* = 0.011).

**Conclusion:**

ICDs are common in EOPD, particularly when associated with the use of dopamine agonists.

## Introduction

Impulse control disorders (ICDs) (compulsive gambling, shopping, hypersexuality, and binge eating behaviors) are defined as excessive and repetitive behaviors that may affect Parkinson’s disease (PD) patients exposed to dopamine agonists (1, 2). These behaviors, along with other ICD-related disorders (i.e., punding, dopamine dysregulation syndrome, walkabouts, compulsive hoarding) can negatively impact patients’ lives, affect their families, and worsen the overall caregiver burden (2–4).

Dopamine agonists are often chosen as the first line therapy especially in young patients to allegedly delay the onset of levodopa-induced motor complications such as dyskinesias. However, their use is associated with a 2–3.5 fold increased risk of developing ICDs, with an overall prevalence of up to 17% in PD patients treated with a dopamine agonist in the largest cohort study (1).

Younger age of PD onset, male gender, higher doses of dopamine agonists, longer disease duration, psychiatric comorbidities (e.g., depression, anxiety), and personal or family history of impulsivity traits were reported to be significant risk factors for the development of ICDs in PD patients (1, 2).

Few reports are available regarding the frequency and characteristics of ICDs in early-onset Parkinson’s disease (EOPD) defined as PD with onset of symptoms before the age of 50 (3). Thus, in this study we aim to assess the frequency of use of dopamine agonists, the prevalence of ICDs, and to explore potential factors associated with their development in a cohort of patients with EOPD.

## Methods

### Patients and diagnosis ascertainment

We used the Mayo Clinic Data Explorer system to identify all patients who received a diagnosis of Parkinsonism between 1990 and 2022. We ascertained potential cases of Parkinsonism using a first computerized screening phase and a second clinical confirmation phase. In phase 1, we searched the indexes for 33 diagnostic codes potentially indicative of Parkinsonism including 5 codes for PD, 14 for Parkinsonism, 7 for tremor, 2 for extrapyramidal disorders, 5 for non-specific neurodegenerative diseases. These 33 codes [see (4)] were the smallest subset of codes that completely captured all cases of Parkinsonism in a previous study of the incidence of Parkinsonism performed in the Olmsted County population (5). This list of 33 codes was designed to yield maximum sensitivity at the cost of low specificity.

In phase 2, a movement disorders specialist (R.S.) reviewed the charts of all patients who had been initially identified as possible Parkinsonism, and who received at least one diagnostic code during the study period. Onset of PD was defined as the approximate date in which at least two of the three cardinal signs of PD were first noted by the patient, by family members or by a healthcare provider as documented in the medical record. The presence of impaired postural reflexes at symptom onset was considered a red flag for a possible alternative diagnosis (6), and those patients who developed abnormal postural reflexes within 3 years of symptoms onset were excluded from this study. The validity of this approach is discussed elsewhere (5). Only those patients with symptoms onset before or at 50 years of age were classified as having EOPD and were included in the study. The medical charts of the EOPD patients were then reviewed to include a list of medications used during the disease course, and to ascertain the presence of ICDs. A patient was diagnosed as having developed ICDs either if this was specifically stated in the chart and supported by appropriate clinical documentation, or if reported in the item *Features of dopamine dysregulation syndrome item of the* Movement Disorder Society Revision of the Unified Parkinson’s Disease Rating Scale (MDS-UPDRS) (7). Cognitive impairments were classified as present only if specifically stated in the patient’s chart, and supported by documented Short Test of Mental Status (STMS) and/or formal neuropsychometric testings (8). The definition of mild cognitive impairment (MCI) and dementia was assessed by using published criteria (9–11). Additional demographic and clinical features relevant to the study questions were abstracted from the medical records.

### Statistical analysis

Numeric features were summarized with medians and interquartile ranges (IQRs); categorical features were summarized with frequency counts and percentages. Patient demographics and disease characteristics were compared between males and females with EOPD as well as between EOPD patients who developed ICD and those who did not, using Wilcoxon rank-sum tests (numeric) and Chi-squared or Fisher’s exact tests (categorical). All tests were two-sided and *p*-values less than 0.05 were considered significant. Analyses were conducted using R version 4.2.2.

### Standard protocol approvals, registrations, and patient consents

This study was approved by the Mayo Clinic and Olmsted Medical Center Institutional Review Boards. Participating patients (or their legally authorized representatives) provided informed written consent for use of their medical information for research.

## Results

A total of 831 patients with EOPD were identified; of these, 513 were males (61.7%). The median age of symptom onset was 42 years (CI: 37–46). No information about the duration of the disease was available for our patients. A family history of PD in a first degree relative was present in 114 (13.7%). A pathogenic gene variant was identified in 11 of the 44 patients who underwent genetic testing (25.0%) (4 PARK2, 3 GBA1, 1 MAPT, 1 LRRK2, 1 PLA2G6, 1 PARK6); however, none of the patients who developed ICD had a positive genetic result. Additional information regarding the demographic and clinical characteristics of these patients are reported in Tables 1, 2.

**Table 1:** Patient demographics and clinical characteristics.

	Female (*N* = 318)	Male (*N* = 513)	Overall (*N* = 831)
Age at symptom onset, years	42 (38, 45)	42 (37, 46)	42 (37, 46)
Age at disease diagnosis, years	46 (42, 49)	46 (42, 49)	46 (42, 49)
Symptom onset to diagnosis, years	2.0 (1.5, 4.0)	3.0 (1.5, 4.0)	2.5 (1.5, 4.0)
Cardinal motor symptoms, n (%)			
Rest tremors	266 (83.6%)	437 (85.2%)	703 (84.6%)
Bradykinesia	264 (83.0%)	438 (85.4%)	702 (84.5%)
Rigidity	251 (78.9%)	404 (79.1%)	655 (79.0%)
Impaired postural reflexes >3 years from symptoms onset	47 (14.8%)	61 (11.9%)	108 (13.0%)
Cognitive impairment post-diagnosis, n (%)			
Mild cognitive impairment (MCI)	14 (4.4%)	36 (7.0%)	50 (6.0%)
Dementia	5 (1.6%)	26 (5.1%)	31 (3.7%)
Dyskinesias, n (%)			
Mild	73 (23.0%)	72 (14.0%)	145 (17.4%)
Moderate	26 (8.2%)	39 (7.6%)	65 (7.8%)
Severe	12 (3.8%)	21 (4.1%)	33 (4.0%)
Diagnosis to dyskinesia, years	6.0 (4.0, 10.0)	6.5 (4.0, 11.0)	6.0 (4.0, 10.0)
Family history of PD, n (%)	50 (15.7%)	64 (12.5%)	114 (13.7%)
Genetic testing for PD, n (%)			
No testing	296 (93.1%)	491 (95.7%)	787 (94.7%)
Negative	14 (4.4%)	19 (3.7%)	33 (4.0%)
Positive	8 (2.5%)	3 (0.6%)	11 (1.3%)
Dopamine agonist treatment, n (%)	154 (48.4%)	259 (50.5%)	413 (49.7%)
Development of impulse control disorder, n (%)	20 (13.0%)	40 (15.4%)	60 (14.5%)
Deep brain stimulation (DBS) treatment, n (%)	56 (17.6%)	96 (18.7%)	152 (18.3%)
Diagnosis to DBS, years	10.0 (7.0, 13.0)	10.0 (7.0, 14.0)	10.0 (7.0, 14.0)

**Table 2:** Clinical characteristics of patients with and without ICD.

	No ICD (*N* = 771)	ICD (*N* = 60)	*P*-value
Age at PD symptom onset, years	42 (37, 46)	40 (35, 55)	0.055
Cardinal motor symptoms, n (%)			
Rest tremors	650 (84.3%)	53 (99.3%)	0.58
Bradykinesia	647 (83.9%)	55 (91.7%)	0.14
Rigidity	607 (78.7%)	48 (82.8%)	0.62
Impaired postural reflexes >3 years from symptoms onset	100 (13.0%)	8 (13.6%)	0.84
Treatment with dopamine agonists, n (%)	355 (46.0%)	60 (100%)	–
Treatment with DA and not levodopa, n (%)	55 (15.5%)	58 (96.7%)	< 0.001*
Cognitive impairment post-PD diagnosis, n (%)			
Mild cognitive impairment (MCI)	46 (6.0%)	4 (6.7%)	0.78
Dementia	23 (3.0%)	8 (13.3%)	< 0.001*
Dyskinesia post-PD diagnosis, n (%)			
Mild	138 (17.9%)	7 (11.7%)	0.36
Moderate	59 (7.7%)	6 (10.0%)	
Severe	29 (3.8%)	4 (6.7%)	
Diagnosis to dyskinesia, years	6.0 (4.0, 10.0)	11.0 (7.0, 16.0)	0.005*
Family history of PD, n (%)	107 (13.9%)	7 (11.7%)	0.85
Genetic testing for PD, n (%)			
No testing	735 (95.3%)	52 (86.7%)	–
Negative	25 (3.2%)	8 (13.3%)	0.17
Positive	11 (1.4%)	0 (0%)	
Deep brain stimulation (DBS) treatment, n (%)	138 (17.9%)	14 (45.2%)	< 0.001 *
Diagnosis to DBS, years	10.0 (7.0, 13.0)	12.0 (10.0, 17.0)	0.079

Dopamine agonists were used in 415/831 (49.9%) patients to treat parkinsonian symptoms at any time during the disease course; of these patients, 60 (14.5%) developed ICDs. A slightly higher prevalence of ICDs was observed in males compared to females (15.4% vs. 13.0% respectively); however, this was not statistically significant (*p* = 0.59). Fifty-eight of these 60 patients (96.7%) were treated with a dopamine agonist monotherapy, whereas 2 received a combination of dopamine agonists plus oral levodopa.

**GRAPH 1:**
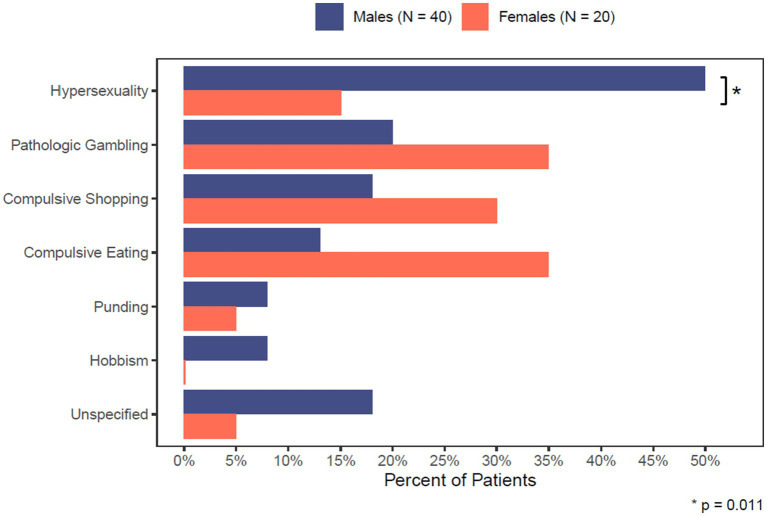
Comparison of ICD sub-types between males and females.

Among the different types of ICDs, hypersexuality was the most commonly identified (38.3%), and it was significantly more frequent in men (*p* < 0.011) (see Table 3; Graph 1). ICDs resolved in 40/60 (67.0%) patients after either decreasing or discontinuing the use of dopamine agonist.

**Table 3:** Frequency of ICD subtypes in males and females.

	Overall (*N* = 60)	Males (*N* = 40)	Females (*N* = 20)	*P*-value
ICD behavior, n (%)				
Pathologic gambling	15 (25%)	8 (20%)	7 (35%)	0.22
Hypersexuality	23 (38%)	20 (50%)	3 (15%)	0.011 *
Compulsive shopping	13 (22%)	7 (18%)	6 (30%)	0.33
Compulsive eating	12 (20%)	5 (13%)	7 (35%)	0.083
Hobbism	3 (5%)	3 (8%)	0 (0%)	0.54
Punding	4 (7%)	3 (8%)	1 (5%)	0.99
Unspecified	8 (13%)	7 (18%)	1 (5%)	0.25

Information about the type and dose of the dopamine agonist used was available for 41/60 patients (68.3%). The most used dopamine agonist was Pramipexole (60.9%), followed by Ropinirole (31.7%), then Rotigotine (7.3%) (Table 4).

**Table 4:** Dosages and levodopa equivalent daily dose of dopamine agonists used in our population.

Dopamine agonist name	Patients (*N* = 49)	Median daily dose in mg (range)	Median LEDD in mg
Pramipexole	25	4.5 (0.375–7.5)	450
Ropinirole	13	12 (2–21)	240
Rotigotine	3	8 (4–8)	240

Levodopa equivalent daily dose (LEDD) was calculated according to the following formula: levodopa 100 mg = Ropinirole 5 mg = Rotigotine 3.3 mg = Pramipexole 1 mg (12).

Patients who developed ICDs had younger PD age of onset compared to those who did not; however, this was not statistically significant (*p* = 0.055). ICD patients were more likely to develop dementia over time (*p* < 0.001). Following the onset of ICDs, the dopamine agonist dose was reduced in 40/60 (0.66%) patients, leading to improvement or resolution of ICDs in all of them.

Dyskinesias were relatively rare in our cohort (17.4%) of EOPD; no significant differences in the overall prevalence of dyskinesias were observed between patients who did and those who did not develop ICDs (*p* = 0.36). However, when dyskinesias were present they tended to develop significantly later in the group of patients that did develop ICDs (median 11.0 vs. 6.0 years after motor symptoms onset respectively, *p* < 0.005) (Table 2).

There were 355 patients in our cohort who used dopamine agonists and did not develop ICDs. Of these, 55 used dopamine agonists as monotherapy, whereas 300 patients used both dopamine agonists and levodopa in combination. Among those 55 patients who used dopamine agonists and did not develop ICDs 29 were male (52.7%) and 26 (47.3%) were female; the median age at PD symptom onset for these patients was 42 (IQR range: 40, 45) years of age. No information regarding the type and dose of dopamine agonist used, and the duration of therapy was available for these patients.

## Discussion

The timing of initiation of symptomatic treatment, and the drug of choice in PD have been a matter of debate for decades. Dopamine agonists are often used as first-line therapy in young patients or in the early stages of disease to limit the risk of inducing motor fluctuations and dyskinesias; however, their use is not short of side effects and they are not nearly as efficacious in treating parkinsonian symptoms as levodopa.

ICDs are a relatively common complication in PD patients treated with dopamine agonists, and younger age of onset of PD, among others, is considered a possible risk factor for their development (1). Although not statistically significant, those who developed ICDs in our cohort of EOPD were younger than those who did not (*p* = 0.055).

The frequency of ICDs in EOPD was previously assessed only by a limited number of studies; an overall prevalence of ICDs of 14.5% was reported in our cohort. The relatively older age of PD onset in our population can explain the similar frequency (17%) that was reported in the largest study published thus far (1); on the other hand, it is lower than the 26.5% reported in a European study that only included patients younger than 40 years of age (13).

When the presence of ICDs was assessed by using the Questionnaire for Impulse Control Disorders in Parkinson’s Disease (QUIP) in patients younger than 45 years old treated with either levodopa or dopamine agonists, a high frequency of around 58% was found in PD patients along with 33% in age and sex-matched controls. This relatively high frequency could potentially be explained by the screening tools used in this study to ascertain the presence of ICDs. While the QUIP is in fact a well validated screening tool with high sensitivity and specificity for ICDs, its relatively low positive predictive value (14) may explain their high results, as supported by the relatively high frequency of ICDs observed in unaffected controls (33%) in the same study (15).

The preferential affinity of the commonly used dopamine agonists for the D3 receptors in the limbic system (16–18) has been for long considered to be the main cause of the onset of these abnormal behaviors in PD patients. However, the majority of patients treated with this class of medications never develops ICDs, suggesting the presence of alternative risk factors for their onset (19). Younger individuals may be more predisposed to developing ICDs due to inherently present impulsive traits associated with their age, regardless of the underlying presence of PD (19). However, additional factors including psychiatric comorbidities and a family history of also holism have been reported as well (19). Sex differences in the type of ICDs developed by PD patients have been reported, with men presenting with excessive gambling and hypersexuality more often than women (1). We observed a slightly higher prevalence of ICDs in our male population, with hypersexuality being the most frequent ICD observed, likely mirroring similar patterns in the general population (20).

While there are no approved medications to treat ICDs, dopamine agonist cessation or dose reduction are usually effective (21); meanwhile, initiation or a careful increase in the levodopa dose may be recommended to avoid low dopaminergic states (i.e., OFF periods). Medication adjustments led to improvement or resolution of ICDs in about 2/3 of patients in our cohort, proving once again the strict neuro-anatomical and pharmacologic relationships between this class of drugs and the onset of these pathologic behaviors (19). Pramipexole and Ropinirole, the two most commonly used dopamine agonists in our cohort, have a high affinity to the D3 dopamine receptors, which are particularly present in the mesolimbic system; this could potentially explain why these reward-seeking behaviors are much more common with the use of this class of drugs than in patients treated with levodopa, which has a lesser affinity to the same receptors (18, 19).

ICDs improvement has been reported in patients undergoing Deep Brain Stimulation (DBS) of the subthalamic nucleus (STN), possibly following a reduction in the dopaminergic drugs; however, a direct effect of the stimulation has also been implicated as a possible explanation (22, 23). This concept has been challenged by the observation that ICDs can at times be worsened or induced following STN DBS (24, 25). None of the 14 ICDs patients who underwent STN DBS exhibited worsening of their symptoms, or developed pathologic impulse control following the surgery.

Frequency and severity of ICDs is higher in Parkin and GBA PD patients (26, 27); this association seems only partly related to the relatively young age of PD onset in this patient population; some have in fact suggested a possible synergistic effect between dopamine agonists and specific mutations (i.e., GBA) cautioning against the use of this class of drugs in these patients (27). It is not surprising that we did not find a genetic mutation in any of our EOPD patients who developed ICDs considering our relatively low sample size, and that Genetic mutations in the genes associated with PD are estimated to be between 10 and 20% in EOPD (28).

Dyskinesias were similarly present in ICDs and non-ICDs patients in our cohort, supporting the presence of overlapping mechanisms in the onset of levodopa-induced dyskinesias and ICDs (29) with an increased probability of co-occurrence of these two disorders (30, 31).

Dopaminergic therapy can influence endogenous dopamine function by interfering with the tonic and phasic activity of the dopaminergic neurons, leading to post-synaptic changes in the expression, density, and activity of dopamine receptors (29); these factors may play a role in the onset of both ICDs and levodopa-induced dyskinesias (29). Similarly, a reduced concentration of striatal dopamine transporters has been well documented in PD patients with ICDs (32, 33), potentially leading to accumulation of dopamine at the synaptic level; this, along with a prolonged duration of action of dopamine may be a common factor for the onset of both ICDs and dyskinesias (29).

Similarly to other studies, patients with dementia were more likely to develop ICDs compared to those without cognitive impairments (34). Executive and decision-making dysfunctions are common in PD patients with and without ICDs (35), with functional imaging studies showing a predominant involvement of the orbitofrontal and anterior cingulate cortices, suggesting a possible common underlying anatomical substrate for the onset of ICDs and dementia in PD patients (35).

Our study has several limitations. First, the overall frequency of ICDs in our cohort may be underrepresented as patients may have been lost at follow up given the tertiary referral center nature of Mayo Clinic. Physicians’ awareness of ICDs has significantly changed over time, which may have influenced the number of cases captured especially in the first years of the analyzed timeframe, which spans between 1990 and 2022. Second, some information, including factors that may have influenced the development of ICDs (e.g., history of alcohol abuse of family history of ICDs), or the time between dopamine agonist initiation and ICDs onset may have not been available to review given the retrospective nature of the study; this may have limited our analysis and influenced some of the results. Third, no validated screening tools (e.g., QUIP) were used to assess the presence of ICDs in our cohort, which may have led to an under representation of ICDs in our cohort. Fourth, we acknowledge the relatively low frequency of genetic tests performed in our population of EOPD patients; genetic tests including only a limited number of pathogenic genes in addition to commercially available genetic tests were at times performed by patients prior to their evaluation at our Institution, limiting our ability to obtain additional genetic testing in a cost effective manner. Fifth, due to the retrospective nature of our study, we were not able to assess the prevalence of ICD in those patients who were not on dopamine agonists; additionally, we were not able to assess the characteristics of those patients who did not develop ICDs even though they used dopamine agonists at any time during their disease course.

## Conclusion

ICDs are common in EOPD, particularly when associated with the use of dopamine agonists, likely due to their higher D3 dopamine receptor affinity in the mesolimbic pathway. ICDs seem to be more common in men than in women; however, when they are present, a reduction in the overall dopamine dose or discontinuation of the offending agent, if possible, lead to substantial improvements. The association between ICDs and the presence of cognitive impairments may have a common neuro-anatomical substrate, and should be evaluated with additional studies.

## Data availability statement

The raw data supporting the conclusions of this article will be made available by the authors, without undue reservation.

## Ethics statement

The studies involving humans were approved by the Mayo Clinic Institutional review board. The studies were conducted in accordance with the local legislation and institutional requirements. Written informed consent for participation was not required from the participants or the participants’ legal guardians/next of kin in accordance with the national legislation and institutional requirements.

## Author contributions

PT: Conceptualization, Writing – original draft, Writing – review & editing. JJ: Writing – review & editing. KG: Writing – review & editing. AM: Data curation, Formal analysis, Writing – review & editing. EC: Writing – review & editing. CS: Writing – review & editing. CP: Writing – review & editing. JB: Writing – review & editing. RS: Conceptualization, Funding acquisition, Writing – review & editing.

## References

[1] Weintraub D Koester J Potenza MN Siderowf AD Stacy M Voon V. Impulse control disorders in Parkinson disease: a cross-sectional study of 3090 patients. Arch Neurol. (2010) 67:589–95. doi: 10.1001/archneurol.2010.65, PMID: 20457959

[2] Faouzi J Corvol JC Mariani LL. Impulse control disorders and related behaviors in Parkinson's disease: risk factors, clinical and genetic aspects, and management. Curr Opin Neurol. (2021) 34:547–55. doi: 10.1097/WCO.0000000000000955, PMID: 33967198

[3] Mehanna R Smilowska K Fleisher J Post B Hatano T Pimentel Piemonte ME. Age cutoff for early-onset Parkinson's disease: recommendations from the International Parkinson and Movement Disorder Society task force on early onset Parkinson's disease. Mov Disord Clin Pract. (2022) 9:869–78. doi: 10.1002/mdc3.13523, PMID: 36247919 PMC9547138

[4] Savica R Grossardt BR Bower JH Ahlskog JE Rocca WA. Incidence and pathology of synucleinopathies and tauopathies related to parkinsonism. JAMA Neurol. (2013) 70:859–66. doi: 10.1001/jamaneurol.2013.114, PMID: 23689920 PMC3707980

[5] Bower JH Maraganore DM McDonnell SK Rocca WA. Incidence and distribution of parkinsonism in Olmsted County, Minnesota, 1976-1990. Neurology. (1999) 52:1214–20. doi: 10.1212/WNL.52.6.1214, PMID: 10214746

[6] Postuma RB Berg D Stern M Poewe W Olanow CW Oertel W. MDS clinical diagnostic criteria for Parkinson's disease. Mov Disord. (2015) 30:1591–601. doi: 10.1002/mds.26424 26474316

[7] Goetz CG Tilley BC Shaftman SR Stebbins GT Fahn S Martinez-Martin P. Movement Disorder Society-sponsored revision of the unified Parkinson's disease rating scale (MDS-UPDRS): scale presentation and clinimetric testing results. Mov Disord. (2008) 23:2129–70. doi: 10.1002/mds.22340, PMID: 19025984

[8] Tang-Wai DF Knopman DS Geda YE Edland SD Smith GE Ivnik RJ. Comparison of the short test of mental status and the mini-mental state examination in mild cognitive impairment. Arch Neurol. (2003) 60:1777–81. doi: 10.1001/archneur.60.12.1777 14676056

[9] Petersen RC. Mild cognitive impairment as a diagnostic entity. J Intern Med. (2004) 256:183–94. doi: 10.1111/j.1365-2796.2004.01388.x 15324362

[10] McKeith IG Galasko D Kosaka K Perry EK Dickson DW Hansen LA. Consensus guidelines for the clinical and pathologic diagnosis of dementia with Lewy bodies (DLB): report of the consortium on DLB international workshop. Neurology. (1996) 47:1113–24. doi: 10.1212/WNL.47.5.1113, PMID: 8909416

[11] Emre M Aarsland D Brown R Burn DJ Duyckaerts C Mizuno Y. Clinical diagnostic criteria for dementia associated with Parkinson's disease. Mov Disord. (2007) 22:1689–707. quiz 1837. doi: 10.1002/mds.21507 17542011

[12] Tomlinson CL Stowe R Patel S Rick C Gray R Clarke CE. Systematic review of levodopa dose equivalency reporting in Parkinson's disease. Mov Disord. (2010) 25:2649–53. doi: 10.1002/mds.23429, PMID: 21069833

[13] Gescheidt T Losada VILY Menšíková K Dušek L Czekóová K Menclová P. Impulse control disorders in patients with young-onset Parkinson’s disease: a cross-sectional study seeking associated factors. Basal Ganglia. (2016) 6:197–205. doi: 10.1016/j.baga.2016.09.001

[14] Weintraub D Hoops S Shea JA Lyons KE Pahwa R Driver-Dunckley ED. Validation of the questionnaire for impulsive-compulsive disorders in Parkinson's disease. Mov Disord. (2009) 24:1461–7. doi: 10.1002/mds.22571, PMID: 19452562 PMC2848971

[15] Vela L Martínez Castrillo JC García Ruiz P Gasca-Salas C Macías Macías Y Pérez Fernández E. The high prevalence of impulse control behaviors in patients with early-onset Parkinson's disease: a cross-sectional multicenter study. J Neurol Sci. (2016) 368:150–4. doi: 10.1016/j.jns.2016.07.003 27538621

[16] Gerlach M Double K Arzberger T Leblhuber F Tatschner T Riederer P. Dopamine receptor agonists in current clinical use: comparative dopamine receptor binding profiles defined in the human striatum. J Neural Transm (Vienna). (2003) 110:1119–27. doi: 10.1007/s00702-003-0027-5, PMID: 14523624

[17] Sokoloff P Giros B Martres MP Bouthenet ML Schwartz JC. Molecular cloning and characterization of a novel dopamine receptor (D3) as a target for neuroleptics. Nature. (1990) 347:146–51. doi: 10.1038/347146a0, PMID: 1975644

[18] Gurevich EV Joyce JN. Distribution of dopamine D3 receptor expressing neurons in the human forebrain: comparison with D2 receptor expressing neurons. Neuropsychopharmacology. (1999) 20:60–80. doi: 10.1016/S0893-133X(98)00066-9 9885786

[19] Ahlskog JE. Pathological behaviors provoked by dopamine agonist therapy of Parkinson's disease. Physiol Behav. (2011) 104:168–72. doi: 10.1016/j.physbeh.2011.04.055, PMID: 21557955

[20] Kuzma JM Black DW. Epidemiology, prevalence, and natural history of compulsive sexual behavior. Psychiatr Clin North Am. (2008) 31:603–11. doi: 10.1016/j.psc.2008.06.005 18996301

[21] Mamikonyan E Siderowf AD Duda JE Potenza MN Horn S Stern MB. Long-term follow-up of impulse control disorders in Parkinson's disease. Mov Disord. (2008) 23:75–80. doi: 10.1002/mds.21770, PMID: 17960796 PMC2651355

[22] Lhommée E Klinger H Thobois S Schmitt E Ardouin C Bichon A. Subthalamic stimulation in Parkinson's disease: restoring the balance of motivated behaviours. Brain. (2012) 135:1463–77. doi: 10.1093/brain/aws078, PMID: 22508959

[23] Lhommée E Wojtecki L Czernecki V Witt K Maier F Tonder L. Behavioural outcomes of subthalamic stimulation and medical therapy versus medical therapy alone for Parkinson's disease with early motor complications (EARLYSTIM trial): secondary analysis of an open-label randomised trial. Lancet Neurol. (2018) 17:223–31. doi: 10.1016/S1474-4422(18)30035-8, PMID: 29452685

[24] Amami P Dekker I Piacentini S Ferré F Romito LM Franzini A. Impulse control behaviours in patients with Parkinson's disease after subthalamic deep brain stimulation: de novo cases and 3-year follow-up. J Neurol Neurosurg Psychiatry. (2015) 86:562–4. doi: 10.1136/jnnp-2013-307214, PMID: 25012201

[25] Kasemsuk C Oyama G Hattori N. Management of impulse control disorders with deep brain stimulation: a double-edged sword. J Neurol Sci. (2017) 374:63–8. doi: 10.1016/j.jns.2017.01.019, PMID: 28126343

[26] Morgante F Fasano A Ginevrino M Petrucci S Ricciardi L Bove F. Impulsive-compulsive behaviors in Parkin-associated Parkinson disease. Neurology. (2016) 87:1436–41. doi: 10.1212/WNL.0000000000003177 27590295 PMC5075971

[27] Amami P de Santis T Invernizzi F Garavaglia B Albanese A. Impulse control behavior in GBA-mutated parkinsonian patients. J Neurol Sci. (2021) 421:117291. doi: 10.1016/j.jns.2020.117291, PMID: 33383316

[28] Schrag A Schott JM. Epidemiological, clinical, and genetic characteristics of early-onset parkinsonism. Lancet Neurol. (2006) 5:355–63. doi: 10.1016/S1474-4422(06)70411-2, PMID: 16545752

[29] Voon V Napier TC Frank MJ Sgambato-Faure V Grace AA Rodriguez-Oroz M. Impulse control disorders and levodopa-induced dyskinesias in Parkinson's disease: an update. Lancet Neurol. (2017) 16:238–50. doi: 10.1016/S1474-4422(17)30004-2, PMID: 28229895

[30] Voon V Sohr M Lang AE Potenza MN Siderowf AD Whetteckey J. Impulse control disorders in Parkinson disease: a multicenter case--control study. Ann Neurol. (2011) 69:986–96. doi: 10.1002/ana.22356, PMID: 21416496

[31] Evans AH Katzenschlager R Paviour D O'Sullivan JD Appel S Lawrence AD. Punding in Parkinson's disease: its relation to the dopamine dysregulation syndrome. Mov Disord. (2004) 19:397–405. doi: 10.1002/mds.20045, PMID: 15077237

[32] Cilia R Ko JH Cho SS van Eimeren T Marotta G Pellecchia G. Reduced dopamine transporter density in the ventral striatum of patients with Parkinson's disease and pathological gambling. Neurobiol Dis. (2010) 39:98–104. doi: 10.1016/j.nbd.2010.03.013, PMID: 20338240

[33] Voon V Rizos A Chakravartty R Mulholland N Robinson S Howell NA. Impulse control disorders in Parkinson's disease: decreased striatal dopamine transporter levels. J Neurol Neurosurg Psychiatry. (2014) 85:148–52. doi: 10.1136/jnnp-2013-305395, PMID: 23899625 PMC4031642

[34] Martinez-Martin P Wan YM Ray Chaudhuri K Schrag AE Weintraub D. Impulse control and related behaviors in Parkinson's disease with dementia. Eur J Neurol. (2020) 27:944–50. doi: 10.1111/ene.14169, PMID: 32048392

[35] Callesen MB Scheel-Krüger J Kringelbach ML Møller A. A systematic review of impulse control disorders in Parkinson's disease. J Parkinsons Dis. (2013) 3:105–38. doi: 10.3233/JPD-120165 23938342

